# JNK interacting protein 1 (JIP-1) protects LNCaP prostate cancer cells from growth arrest and apoptosis mediated by 12-*0*-tetradecanoylphorbol-13-acetate (TPA)

**DOI:** 10.1038/sj.bjc.6601834

**Published:** 2004-04-27

**Authors:** T Ikezoe, Y Yang, H Taguchi, H P Koeffler

**Affiliations:** 1 Division of Hematology/Oncology, Cedars-Sinai Research Institute, University of California-Los Angeles School of Medicine, Los Angeles, CA 90048, USA; 2 Department of Internal Medicine, Kochi Medical School, Kochi 783-8505, Japan

**Keywords:** TPA, LNCaP, JNK, JIP-1, c-Jun/AP-1, apoptosis

## Abstract

12-*0*-tetradecanoylphorbol-13-acetate (TPA) stimulates protein kinase C (PKC) which mediates apoptosis in androgen-sensitive LNCaP human prostate cancer cells. The downstream signals of PKC that mediate TPA-induced apoptosis in LNCaP cells are unclear. In this study, we found that TPA activates the c-Jun NH_2_-terminal kinase (JNK)/c-Jun/AP-1 pathway. To explore the possible role that the JNK/c-Jun/AP-1 signal pathway has on TPA-induced apoptosis in LNCaP cells, we stably transfected the scaffold protein, JNK interacting protein 1 (JIP-1), which binds to JNK inhibiting its ability to phosphorylate c-Jun. TPA (10^−9^–10^−7^ mol l^−1^) caused phosphorylation of JNK in both wild-type and JIP-1-transfected (LNCaP-JIP-1) cells. It resulted in phosphorylation and upregulation of expression of c-Jun protein in the wild-type LNCaP cells, but not in the JIP-1-transfected LNCaP cells. In addition, upregulation of AP-1 reporter activity by TPA (10^−9^ mol l^−1^) occurred in LNCaP cells but was abrogated in LNCaP-JIP-1 cells. Thus, TPA stimulated c-Jun through JNK, and JIP-1 effectively blocked JNK. TPA (10^−12^–10^−8^ mol l^−1^) treatment of LNCaP cells caused their growth inhibition, cell cycle arrest, upregulation of p53 and p21^*waf1*^, and induction of apoptosis. All of these effects were significantly attenuated when LNCaP-JIP-1 cells were similarly treated with TPA. A previous study showed that c-Jun/AP-1 blocked androgen receptor (AR) signaling by inhibiting AR binding to AR response elements (AREs) of target genes including prostate-specific antigen (PSA). Therefore, we hypothesised that TPA would not be able to disrupt the AR signal pathway in LNCaP-JIP-1 cells. Contrary to expectation, TPA (10^−9^–10^−8^ mol l^−1^) inhibited DHT-induced AREs reporter activity and decreased levels of PSA in the LNCaP-JIP-1 cells. Taken together, TPA, probably by stimulation of PKC, phosphorylates JNK, which phosphorylates and increases expression of c-Jun leading to AP-1 activity. Growth control of prostate cancer cells can be mediated through the JNK/c-Jun pathway, but androgen responsiveness of these cells can be independent of this pathway, suggesting that androgen independence in progressive prostate cancer may not occur through activation of this pathway.

12-*0*-tetradecanoylphorbol-13-acetate (TPA) exerts a variety of effects on cells that include proliferation, malignant transformation, differentiation, and cell death (Hunter and Karin, 1992). Growth stimulation induced by TPA was shown in fibroblasts, epidermal cells, lymphocytes, and several type of cancer cells including KG-1, a very immature human myeloblastic leukaemia cell line (Koeffler, 1981; Agadir *et al*, 1999); in contrast, TPA induced differentiation and growth arrest of human U937 myelomonocytic and HL-60 myeloblastic leukaemia cells (Rovera *et al*, 1979; Koeffler *et al*, 1981; Gaynor *et al*, 1991; Kaneki *et al*, 1999; Takada *et al*, 1999). Previous studies suggested that upregulation of c-Jun (Gaynor *et al*, 1991), tumour necrosis factor *α* (TNF *α*) (Takada *et al*, 1999), or protein kinase C (PKC) *β* (Kaneki *et al*, 1999) might play an important role in the induction of differentiation and cell growth arrest in these cells. In addition, TPA was shown to induce apoptosis in LNCaP, an androgen-sensitive human prostate cancer cell line through the upregulation of PKC-*δ* (Fujii *et al*, 2000), -*α* (Garzotto *et al*, 1998), or ceramide synthesis (Powell *et al*, 1996). Other studies also showed that TPA upregulated the expression of p21^*waf1*^ and downregulated the levels of c-Myc as growth of LNCaP cells slowed (Mitchell and El-Deiry, 1999).

Through the PKC pathway, TPA phosphorylates c-Jun-NH_2_-terminal protein kinase (JNK), which belongs to the mitogen-activated protein kinase (MAPK) family (Karin, 1995). Phosphorylated JNK rapidly phosphorylates Ser-63 and Ser-73 of the c-Jun amino terminus, resulting in induction of c-Jun synthesis. The activity of c-Jun/AP-1 is regulated by increased synthesis and phosphorylation of c-Jun (Karin, 1995; Davis, 2000). c-Jun-NH_2_-terminal protein kinase has been suggested to enhance the induction of apoptosis; however, the mechanism by which this occurs is unclear. One possibility is that phosphorylated c-Jun causes the release of cytochrome *c* from mitochondria which then acts with Apaf-1 to activate caspase-9 and caspase-3, and orchestrates apoptosis (Behrens *et al*, 1999; Davis, 2000). 12-*0*-tetradecanoylphorbol-13-acetate has been shown to enhance AP-1 transcriptional activity in LNCaP cells (Sato *et al*, 1997); but the contribution of the JNK/c-Jun/AP-1 pathway to TPA-induced apoptosis in LNCaP cells remains to be fully elucidated.

PSA belongs to the kallikrein-like serine protease family. It is produced almost exclusively by the prostate epithelial cells, and is used as a serum marker for diagnosis and progression of prostate cancer (Polascik *et al*, 1999). The 5′ upstream promoter and enhancer region of the *PSA* gene contains several androgen receptor response elements (AREs) to which ligand-activated androgen receptor (AR) binds and induces expression of PSA (Huang *et al*, 1999; Hisatake *et al*, 2000). Previous studies showed that TPA downregulated the expression of PSA without any downregulation of levels of AR in LNCaP cells (Andrews *et al*, 1992; Sato *et al*, 1997). The investigators suggested that c-Jun/AP-1 played an integral role by binding to the DNA-binding domain of AR, disrupting the AR/ARE complex formation (Sato *et al*, 1997).

Recently, two cytoplasmic proteins identified as JNK interacting proteins 1 and 2 (JIP-1/2), were found to bind selectively to JNK but not to other group of MAPKs families including p38 and extracellular signal-regulated protein kinase (ERK) (Dickens *et al*, 1997; Whitmarsh *et al*, 1998; Yasuda *et al*, 1999; Harding *et al*, 2001). Overexpression of JIP-1 caused the cytoplasmic retention of JNK and thereby inhibited the expression of genes mediated by JNK, including c-Jun (Dickens *et al*, 1997; Whitmarsh *et al*, 1998; Yasuda *et al*, 1999; Harding *et al*, 2001). Moreover, recent studies showed that JIP-1 inhibited the biological actions of the JNK signal pathway. Overexpression of JNK-binding domain (JBD) of JIP-1 suppressed the malignant transformation of murine B cells expressing *Bcr/Abl*, an oncogene which activates the JNK signal pathway (Dickens *et al*, 1997). Also, overexpression of JBD of JIP-1 prevented apoptosis of neurons after withdrawal of nerve growth factor (Harding *et al*, 2001).

In this study, we explore the role of the JNK/c-Jun/AP-1 signal pathway in TPA-induced apoptosis and downregulation of PSA in LNCaP cells by stably transfecting JBD of JIP-1 in these cells and studying their biological responses compared to wild-type cells.

## MATERIALS AND METHODS

### Cell culture

LNCaP cells were obtained from American Type Culture Collection (Rockville, MD, USA) and maintained in RPMI 1640 with 10% FCS.

### Chemicals

TPA and JNK inhibitor SP600125 were obtained from Sigma (St Louis, MO, USA) and Calbiochem (San Diego, CA, USA), respectively.

### Soft agar colony assay

Cells were cultured in a two-layer soft agar system for 14 days as previously described (Hisatake *et al*, 1999). Washed, single-cell suspension of cells were enumerated and plated into 24-well flat-bottom plates with a total of 1 × 10^3^ cells well^−1^ in a volume of 400 *μ*l well^−1^. The feeder layer was prepared with agar that had been equilibrated at 42°C. Prior to this step, TPA was pipetted into the wells. After incubation, colonies were counted. Experiments were carried out twice using triplicate plates.

### MTT assay

Cells (10^4^ ml^−1^) were incubated with various concentrations of TPA (10^−10^–10^−8^ mol l^−1^) for 4 days in 96-well plates (Flow Laboratories, Irvine, CA, USA). After culture, cell number and viability were evaluated by measuring the mitochondrial-dependent conversion of the tetrazolium salt, MTT (Sigma), to a colored formazan product. MTT (0.5 mg ml^−1^ in PBS) was added to each well and incubated for 4 h at 37°C. The medium was then carefully aspirated, and dimethyl sulfoxide (DMSO; Burdick & Jackson, Muskegon, MI, USA) was added to solubilise the coloured formazan product. Absorbance was read at 540 nm on a scanning multiwell spectrophotometer (Bio-Rad) after agitating the plates for 5 min on a shaker.

### Cell cycle analysis

Cells were incubated for 24 h either with or without TPA (10^−9^–10^−8^ mol l^−1^). They were fixed in chilled methanol overnight before staining with 50 *μ*l ml^−1^ propidium iodide (PI) in the presence of RNase (Promega, Madison, WI, USA), as described previously (Hisatake *et al*, 1999). Cell cycle status was analysed on FACscan Flow Cytometery and CellFit Cell-Cycle Analysis software.

### Assessment of apoptosis

Apoptotic cell death was examined by terminal deoxyribonucleotide transferase-mediated dUTP nick-end labeling (TUNEL) method using the *In situ* Cell Death Detection kit (Roche Molecular Biochemicals, Germany), according to the manufacture's instruction. For quantification, three different fields were counted under the microscope and at least 300 cells were counted in each field. All experiments were performed twice.

### Plasmids

ARE4-E4 Lux, which is the multimerised four consensus AREs from the PSA promoter cloned upstream of the luciferase gene in the pGL3 vector (Promega, Chicago, IL), was used (Huang *et al*, 1999; Hisatake *et al*, 2000). TRE-Luc construct was a generous gift from Christopher K Glass (University of California, San Diego, CA, USA) (Ricote *et al*, 1998). The FLAG-tagged JNK-binding domain (JBD) of JIP-1 (residues 127–281) was cloned into a pcDNA3 vector (Clontech, San Francisco, CA, USA) and was a generous gift from Charles L Sawyers (University of California, Los Angeles) (Dickens *et al*, 1997).

### Establishment of stable transfected LNCaP cell line

LNCaP cells were transfected with the JBD-pcDNA3 vector using GenePORTER transfection reagent (Gene Therapy Systems, Inc., San Diego, CA, USA). Selection was performed with 800 *μ*g ml^−1^ G418 (Omega Scientific, Inc., Tarzana, CA, USA). The expression of JIP-1 was confirmed by Western blot analysis using anti-Flag antibody (Sigma).

### Transfections and luciferase assay

LNCaP or LNCaP-JIP-1 cells were plated in 24-well plates and incubated until 60–80% confluency. Cells were transfected with the indicated plasmids using the GenePORTER transfection. Following transfection, cells were incubated with 10% charcoal-stripped FBS RPMI 1640 either with or without DHT (10^−8^ mol l^−1^) and either with or without TPA for various durations. Luciferase activity in cell lysates was measured by Dual Luciferase assay system (Promega, Madison, WI, USA). Luciferase activity was normalised by renilla activity. The results were presented as the fold induction, which is the relative luciferase activity of the treated cells over that of control cells. All transfection experiments were carried out in triplicate wells and repeated separately at least three times.

### Western blot analyses

Cells were seeded on 60 mm plates and incubated until 60–80% confluency, then the medium was replaced with RPMI 1640 containing 10% charcoal-striped FBS either with or without DHT (10^−8^ mol l^−1^) and either with or without TPA. After incubation, cells were washed twice in PBS, and whole-cell lysates were prepared; cells were suspended in lysis buffer (50 mM Tris (pH 8.0), 150 mM NaCl, 0.1% SDS, 0.5% sodium deoxycholate, 1% NP 40, 100 *μ*g ml^−1^ phenylmethysulphonyl fluoride, 1 mM NaF, 1 mM NaVO_3_, 2 *μ*g ml^−1^ aprotinin, 1 *μ*g ml^−1^ pepstatin, and 10 *μ*g ml^−1^ leupeptin), and placed on ice for 30 min. After centrifugation at 15 000 **g** for 20 min at 4°C, the supernatant was collected. Protein concentrations were quantitated using a Bio-Rad assay (Bio-Rad Laboratories, Hercules, CA, USA). Proteins were resolved on a 4–15% SDS polyacrylamide gel, transferred to an immobilon polyvinylidene difuride membrane (Amersham Corp., Arlington Heights, IL, USA), and probed sequentially with a variety of antibodies. Anti-c-Jun (sc-44, Santa Cruz, Santa Cruz, CA, USA), anti-p-c-Jun (KM-1, Santa Cruz), anti-JNK (sc-571, Santa Cruz), anti-p-JNK (sc-6254, Santa Cruz), anti-PSA C-19 (Santa Cruz), p-53 (Santa Cruz), p21^*waf1*^ (Calbiochem, Darmstadt, Germany), and anti-actin antibody (Santa Cruz) were used. The blots were developed using the enhanced chemiluminescence kit (Amersham Corp.).

### Evaluation of DNA-binding activity of AP-1 by enzyme-linked immunosorbent assay (ELISA)

The DNA-binding activity of AP-1 was quantified by ELISA using the Trans-AM AP-1 Transcription Factor Assay kit (Active Motif North America, Carlsbad, CA, USA), according to the instructions of the manufacturer. Briefly, nuclear extracts were prepared as previously described and incubated in 96-well plates coated with immobilised oligonucleotide (5′-CGCTTGATGAGTCAGCCGGAA-3′) containing a consensus (5′-TGAGTCA-3′)-binding site for AP-1. AP-1 binding to the target oligonucleotide was detected by incubation with primary antibody specific for the activated form of c-Jun (Active Motif North America), visualised by anti-IgG horseradish peroxidase conjugate and Developing Solution, and quantified at 450 nm with a reference wavelength of 655 nm. Background binding was subtracted from the value obtained for binding to the consensus DNA sequence.

### Statistical analysis

Statistical analysis was performed by Student's *t*-test.

## RESULTS

### Generation of LNCaP-JIP-1 cells

**Figure 1:**
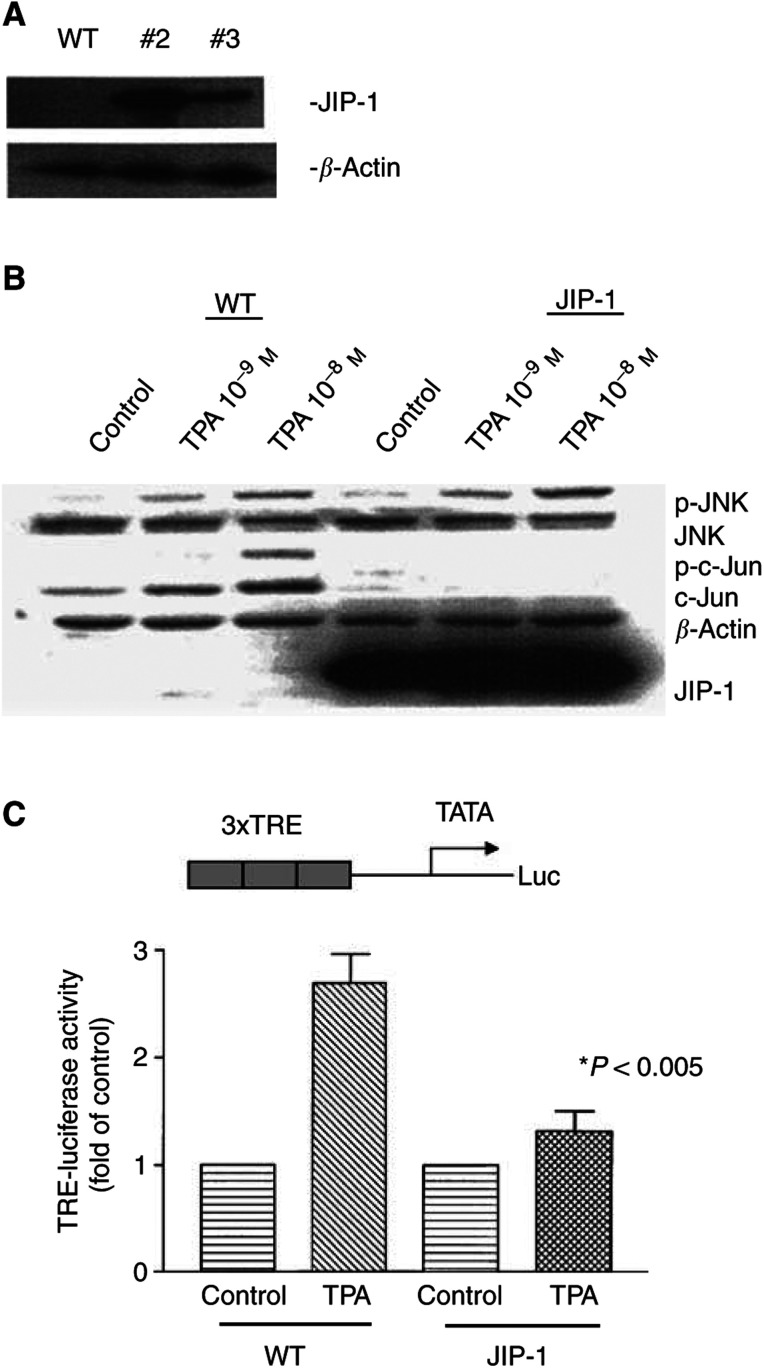
(**A**) Establishment of JIP-1-expressing LNCaP cells. LNCaP cells were transfected with an expression vector encoding the JBD of JIP-1, and G418-resistant clones #2 and #3 were isolated. Proteins were extracted from these cells, and subjected to Western blot analysis. The membrane was sequentially probed with antibodies against Flag and *β*-actin. (**B**) Expression of JIP-1 in LNCaP cells inhibits phosphorylation of Ser-63 of c-Jun and decreases total levels of c-Jun protein. JNK-binding domain of JIP-1 was stably transfected in LNCaP cells. Wild-type LNCaP cells and LNCaP-JIP-1 cells were cultured either with or without TPA (10^−9^, 10^−8^ mol l^−1^) for 18 h, then proteins were extracted and subjected to Western blot analysis. The membrane was sequentially probed with antibodies against phospho-JNK (Thr-183 and Tyr-185), JNK-1, phospho-c-Jun (ser-63), c-Jun, *β*-Actin and Flag. (**C**) Expression of JIP-1 inhibits TPA-induced AP-1 reporter activity in LNCaP cells. The reporter construct (TRE-Luc) is shown at the top. Wild-type or JIP-1 stably expressing LNCaP cells were transfected with the reporter construct (0.8 *μ*g) and cultured either with or without TPA (10^−9^ mol l^−1^) for 6 h. pRL-SV40-Luciferase (Renilla luciferase) vector was cotransfected for normalisation. SDs derived from duplicate experiments with triplicate dishes per point. ^*^, *P*<0.005 as determined by Student's *t*-test differences between wild-type and JIP-1 cells.

LNCaP cells were transfected with an expression vector encoding JBD of JIP-1, and G418-resistant clones #2 and #3 were isolated that stably expressed JBD, detectable with anti-Flag antibody (Figure 1A

). The level of JIP-1 was higher in clone #2 than #3; therefore, clone #2 LNCaP-JIP-1 cells were used in further studies unless indicated.

### JIP blocked TPA-induced phosphorylation and expression of c-Jun as well as AP-1 reporter activity in LNCaP-JIP-1 cells

12-*0*-tetradecanoylphorbol-13-acetate (10^−9^–10^−8^ mol l^−1^, 16 h) caused phosphorylation of JNK in a dose-dependent manner in both wild-type and JIP-1-transfected LNCaP cells (Figure 1B). 12-*0*-tetradecanoylphorbol-13-acetate also increased expression of c-Jun and phosphorylated c-Jun in wild-type LNCaP cells, but not in LNCaP-JIP-1 cells (Figure 1B). To evaluate the influence of expression of JIP-1 upon AP-1 transcriptional activity, reporter assays were performed using an AP-1 luciferase construct, which contained three copies of the TPA response elements (TRE). 12-*0*-tetradecanoylphorbol-13-acetate (10^−9^ mol l^−1^, 6 h) induced AP-1 reporter activity by 2.7-fold in wild-type LNCaP cells; however, in LNCaP-JIP-1 cells, induction was 1.3-fold, compared to untreated LNCaP-JIP-1 cells (Figure 1C). Taken together, these data suggested that JIP-1 effectively blocked TPA-induced c-Jun/AP-1 transcriptional activity.

### JIP-1 protected LNCaP-JIP-1 cells from growth inhibition mediated by TPA

**Figure 2:**
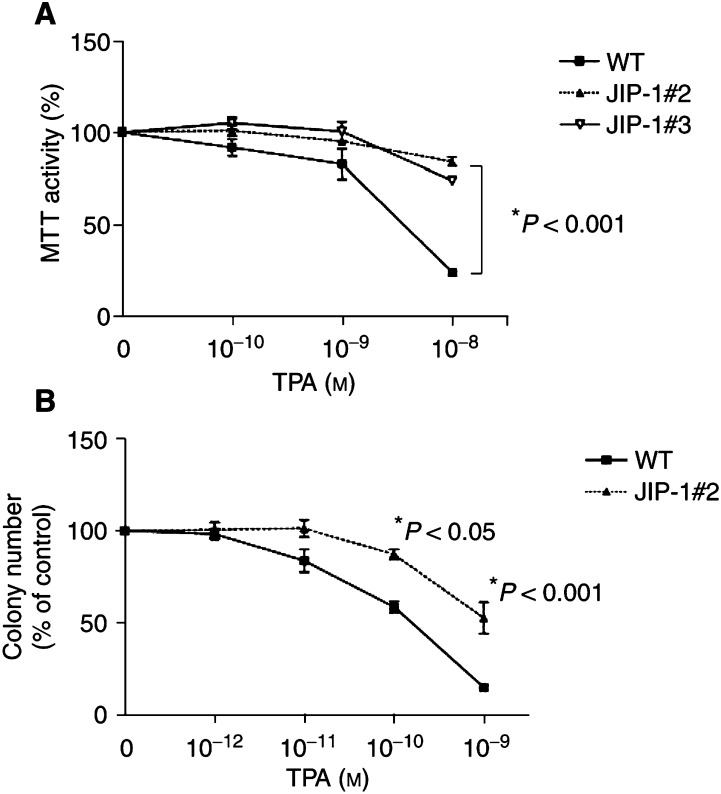
Expression of JIP-1 partially rescues LNCaP cells from TPA-induced growth inhibition. (**A**) MTT assay: Wild-type or JIP-1 stably transfected LNCaP cells were placed in 96-well plates and cultured either with or without TPA (10^−10^–10^−8^ mol l^−1^). After 4 days, the cells were treated with MTT for 4 h, and MTT activity was measured. (**B**) Colony assay: Wild-type or JIP-1 stably transfected LNCaP cells were plated in soft agar either with or without TPA (10^−10^–10^−8^ mol l^−1^), and colonies were enumerated after 14 days of culture. For both series of studies, each point represents a mean of two independent experiments with triplicate dishes for each experimental point; bars, s.d. Results are expressed as mean percentage of either colonies or MTT activity in control plates containing cells, not exposed to TPA but cultured with the same amount of diluent (DMSO). The *P*-value was determined by Student's *t*-test differences between wild-type and JIP-1 cells.

Effect of different concentrations of TPA on cellular progression was measured by several different assays. Growth in liquid culture was measured by MTT assay (described in Materials and Methods section). Almost identical MTT activity was obtained from the untreated control cells of both the wild-type and JIP-1-transfected LNCaP cells (data not shown). In the presence of increasing concentrations of TPA, both clone #2 and #3 LNCaP-JIP-1 cells showed significantly higher MTT activity compared to the wild-type cells (Figure 2A

). For example, TPA (10^−8^ mol l^−1^, 4 days) reduced MTT activity by 75% in the wild-type cells; on the other hand, under similar conditions, only a 15% reduction occurred in the clone #2 LNCaP-JIP-1 population (*P*<0.005). Consistent with these results, colony assays showed that LNCaP-JIP-1 cells were more resistant to increasing concentrations of TPA compared to wild-type cells (Figure 2B). Both wild-type and clone #2 of JIP-1-transfected cells formed approximately 300 colonies in untreated control wells, and colony size was almost the same in both groups. 12-*0*-tetradecanoylphorbol-13-acetate effectively inhibited the clonal growth by 50% (ED_50_) at approximately 1.3 × 10^−10^ mol l^−1^ for the wild-type LNCaP cells and 10^−9^ mol l^−1^ for clone #2 LNCaP-JIP-1 cells; this TPA concentration inhibited clonal growth of wild-type cells by 85% (*P*<0.01) (Figure 2B).

### JNK inhibitor SP600125 protected LNCaP cells from growth inhibition mediated by TPA

**Figure 3:**
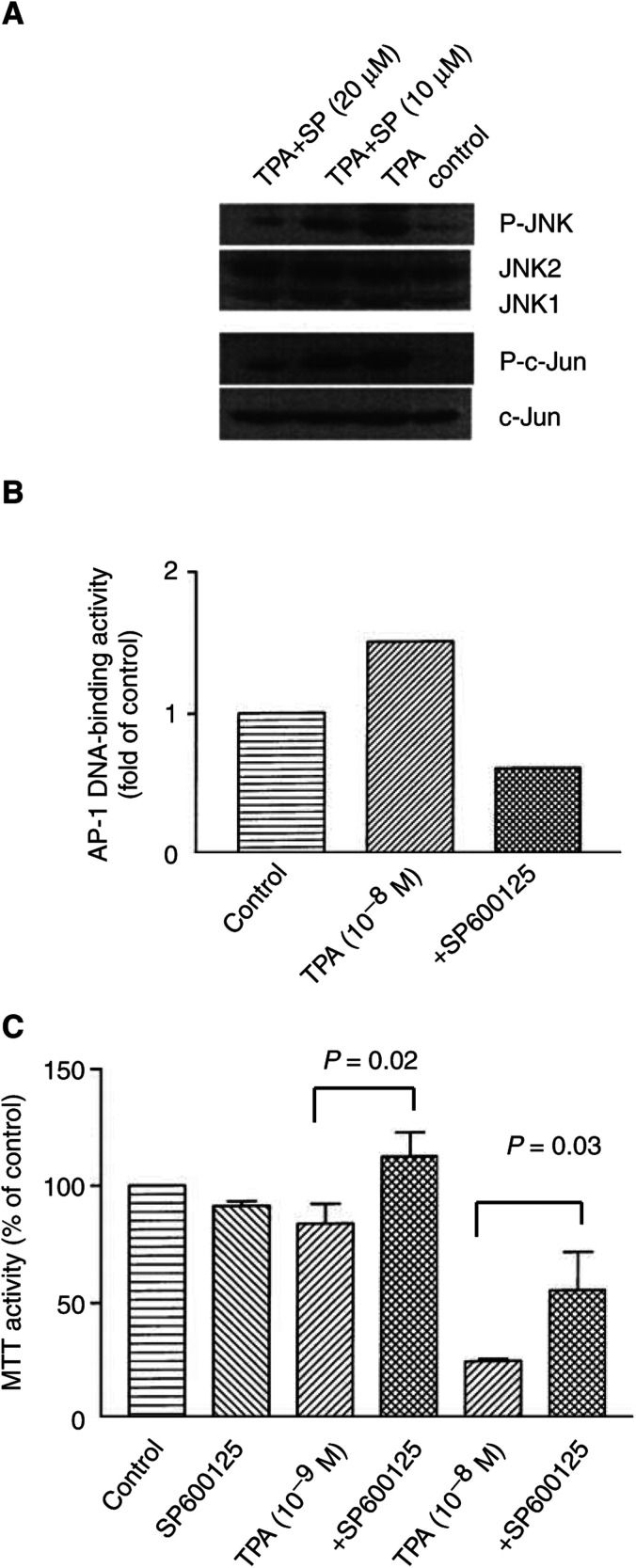
(**A**) SP600125 inhibits TPA-induced phosphorylation of JNK and c-Jun in LNCaP cells. LNCaP cells were cultured with TPA (10^−8^ mol l^−1^) either alone or in combination with SP600125 (10 or 20 *μ*M) for 16 h, then proteins were extracted and subjected to Western blot analysis. The membrane was sequentially probed with antibodies against phospho-JNK (Thr-183 and Tyr-185), JNK-1, phospho-c-Jun (ser-63), and c-Jun. Control cells were cultured in the presence of control diluent. SP, SP600125. (**B**) SP600125 inhibits TPA-induced AP-1/DNA binding activity in LNCaP cells. LNCaP cells were cultured with TPA (10^−8^ mol l^−1^) either alone or in combination with SP600125 (20 *μ*M) for 16 h, then nuclear proteins were extracted and subjected to ELISA to measure DNA-binding activity of AP-1. Control cells were cultured in the presence of control diluent. (**C**) Exposure of LNCaP cells to JNK inhibitor SP600125 partially rescues LNCaP cells from TPA-induced growth inhibition. LNCaP cells were plated in 96-well plates and cultured with TPA (10^−9^ or 10^−8^ mol l^−1^), SP600125 (20 *μ*M) either alone or in combination of both. After 4 days, the cells were treated with MTT for 4 h, and MTT activity was measured.

Furthermore, we cultured LNCaP cells in the presence of the JNK inhibitor SP600125 to block the JNK/c-Jun/AP-1 pathway. As shown in Figure 3A

, exposure of LNCaP cells to SP600125 (10 or 20 *μ*M) effectively downregulated levels of TPA-induced phosphorylation of JNK and c-Jun in a dose-dependent manner. Also, SP600125 completely inhibited TPA-stimulated AP1/DNA-binding activity in these cells, as measured by an ELISA-based assay (Figure 3B). LNCaP cells were cultured with either TPA or SP600125 (20 *μ*M) alone or the combination of both for 4 days. SP600125 alone did not affect the proliferation of LNCaP cells (Figure 3C); SP600125 blunted the ability of TPA to induce growth arrest of LNCaP cells as measured by MTT assay (Figure 3C).

### JIP attenuated TPA-induced apoptosis and cell cycle arrest in LNCaP-JIP-1 cells

**Figure 4:**
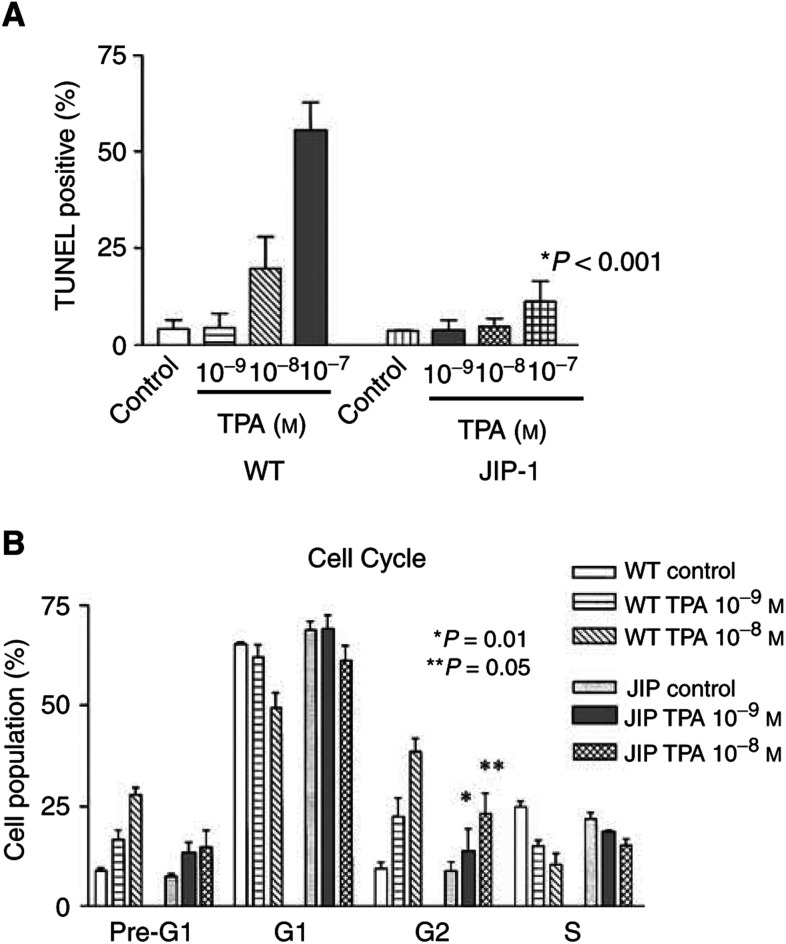
Expression of JIP-1 in LNCaP cells attenuates TPA-induced apoptosis and cell cycle arrest. (**A**) TUNEL assay: Wild-type or JIP-1 stably transfected LNCaP cells were plated in 96-well plates and cultured either with or without TPA (10^−9^–10^−7^ mol l^−1^); and 24 h later, apoptosis was determined by TUNEL assay. Results represent the mean±s.d. of two experiments carried out in triplicates. (**B**) Cell cycle analysis: Wild-type or JIP-1 stably transfected LNCaP cells were plated in 12-well plates and cultured either with or without TPA (10^−9^–10^−8^ mol l^−1^) for 24 h at which time the cell cycle status was analysed. The *P*-values were determined by Student's *t*-test difference between wild-type and JIP-1 cells.

To detect apoptosis, TUNEL assay was performed. As previous studies (Koeffler *et al*, 1981; Kaneki *et al*, 1999) showed, TPA (10^−9^–10^−7^ mol l^−1^, 24 h) induced apoptosis in wild-type LNCaP cells in a dose-dependent manner (Figure 4A

). For example, 10^−8^ and 10^−7^ mol l^−1^ of TPA induced 20±7 and 55±8% of cells to become apoptotic, respectively. In contrast, only 5±2 and 12±5% of LNCaP-JIP-1 cells were apoptotic in the presence of 10^−8^ and 10^−7^ mol l^−1^ TPA, respectively.

Cell cycle analysis showed that untreated wild-type and JIP-1-transfected cells were essentially identically in the percent cells in each phase of the cell cycle (Figure 4B). A difference in these populations of cells emerged in the presence of the phorbol diester. 12-*0*-tetradecanoylphorbol-13-acetate (10^−9^–10^−8^ mol l^−1^, 16 h) induced a G_2_/M cell cycle arrest in the wild-type LNCaP cells in a dose-dependent manner (control, 10±3%; TPA 10^−9^ mol l^−1^, 23±8%; TPA 10^−8^ mol l^−1^, 39±5%). On the other hand, the population of LNCaP-JIP-1 cells in G2/M of the cell cycle was significantly attenuated (control, 9±4%; TPA, 10^−9^ mol l^−1^, 14±8% (*P*=0.01), 10^−8^ mol l^−1^, 23±9% (*P*=0.05)). The appearance of cells with a fractional DNA content (pre-G_0_/G_1_ phase), a feature characteristic of apoptosis, was prominent in wild-type LNCaP cells after their exposure to TPA with nearly twice the number of cells accumulating in the pre-G_0_/G_1_ phase in wild-type LNCaP cells treated with 10^−8^ mol l^−1^ TPA (wild type, 28±3%) as compared to the JIP-1-transfected cells (15±6%).

**Figure 5:**
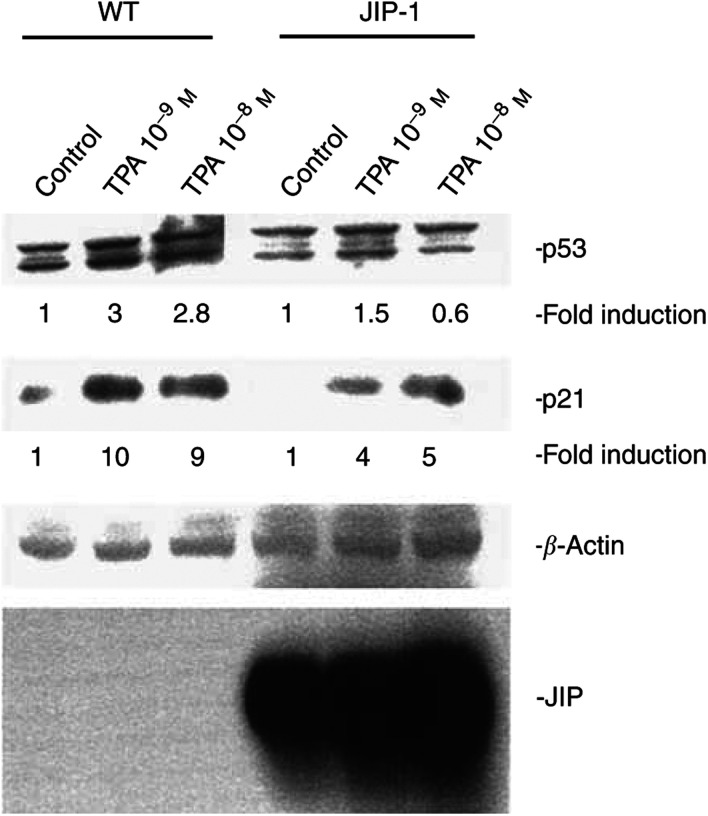
Expression of JIP-1 in LNCaP cells attenuates their TPA-induced expression of p53 and p21^*waf1*^. Wild-type and JIP-1 stably transfected cells were seeded on 60 mm plates and incubated until 60–80% confluency; the medium was replaced with RPMI 1640 containing 10% FBS either with or without TPA (10^−9^–10^−8^ mol l^−1^). After 18 h, cells were harvested. Lysates were made and subjected to Western blot analysis. The band intensities were measured by densitometry.

The cyclin-dependent kinase inhibitor p21^*waf1*^ helps to regulate the cell cycle; previous studies showed that TPA induced p21^*waf1*^ in LNCaP cells associated with slowing of the cell cycle (Garzotto *et al*, 1998). Consistent with the results from the proliferation assays and cell cycle analysis, induction of p21^*waf1*^ by exposure to TPA was attenuated by about 60% in the LNCaP-JIP-1 cells compared to LNCaP cells (Figure 5

). The p53 protein is the upstream regulator of p21^*waf1*^. The TPA-induced elevated levels of p53 were also blunted in the LNCaP-JIP-1 cells by about half compared to LNCaP cells (Figure 5).

### 12-*0*-tetradecanoylphorbol-13-acetate decreases androgen responsiveness in LNCaP cells which is independent of the JNK/c-Jun pathway

**Figure 6:**
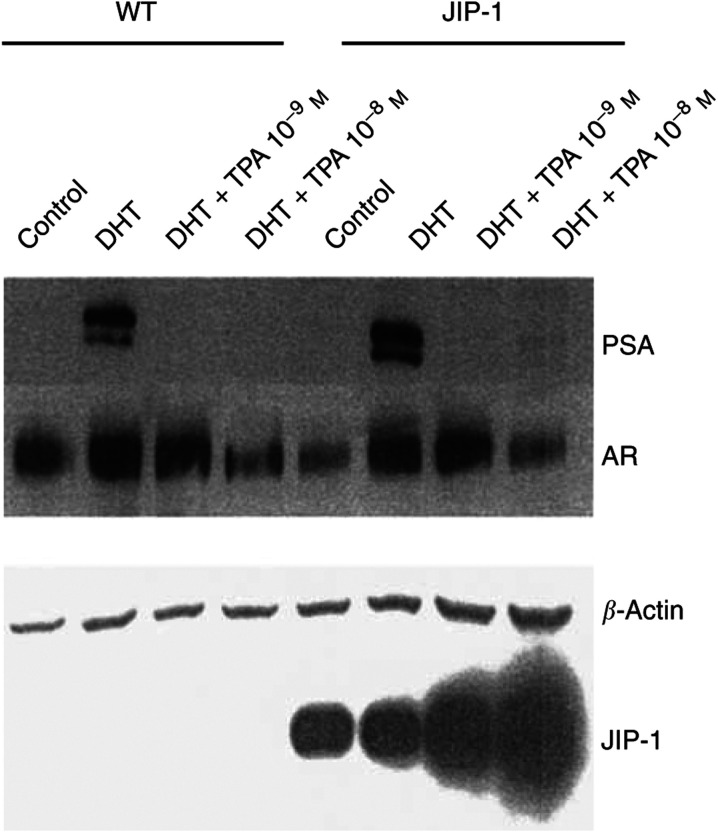
Blockade of c-Jun pathway does not prevent decrease levels of PSA by TPA. Wild-type and JIP-1 stably transfected LNCaP cells at 60% confluency were incubated in culture medium containing 10% charcoal-striped FBS for 24 h before the addition of DHT (10^−8^ mol l^−1^) either with or without TPA (10^−9^–10^−8^ mol l^−1^). Cells were cultured for an additional 18 h. Lysates were made and subjected to Western blotted. Control: cell lysates harvested before the addition of reagents. PSA, prostate-specific antigen; AR, androgen receptor.

We evaluated the responsiveness of prostate cancer cells to androgen when their JNK/c-Jun pathway was blocked, taking advantage of the LNCaP-JIP-1 cells. To evaluate the expression level of PSA in LNCaP-JIP-1 cells treated with DHT±TPA (10^−9^–10^−8^ mol l^−1^), Western blot analysis was performed (Figure 6

). In both LNCaP and LNCaP-JIP-1 cell lines, DHT (10^−8^ mol l^−1^, 18 h) markedly and equally induced the expression of PSA. In wild-type LNCaP cells, TPA (10^−9^ mol l^−1^) completely blocked the DHT-induced expression of PSA without any significant decrease in the cellular level of AR at this dose, which was consistent with previous results (Andrews *et al*, 1992; Sato *et al*, 1997). Even though LNCaP-JIP-1 cells were incapable of a TPA-inducible activation of c-Jun (or phosphorylated c-Jun) (Figure 1B), expression of PSA was completely abrogated similar to what occurred in the wild-type cells (Figure 6).

**Figure 7:**
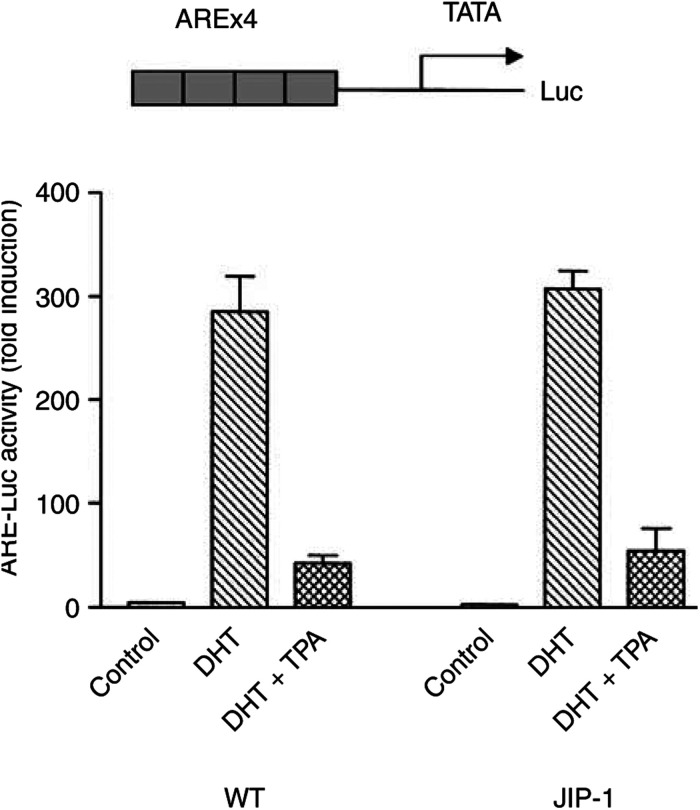
Blockade of Jun pathway does not prevent decrease ARE activation by TPA in LNCaP cells. Shown at the top is the reporter gene (ARE4-E4Lux) containing the four concatmerised androgen receptor response elements (ARE) identical to those in the PSA enhancer, which is attached to the luciferase (Luc) reporter. Wild-type and JIP-1 stably transfected LNCaP cells were transfected with ARE4-E4Lux (0.8 *μ*g). DHT (10^−8^ mol l^−1^) was added either with or without TPA (10^−9^ mol l^−1^). Results represent the mean±s.d. of three experiments with triplicate dishes per experimental point. pRL-SV40-Luciferase (renilla luciferase) vector was cotransfected for normalisation.

Also, ARE-luciferase reporter assays were performed. The LNCaP-JIP-1 cells were cultured with DHT (10^−8^ mol l^−1^) after they were transfected with an ARE-E4-luciferase reporter vector in which ARE I from the *PSA* gene was concatmerised (Huang *et al*, 1999; Hisatake *et al*, 2000). The reporter activity increased about 300-fold after addition of DHT (10^−8^ mol l^−1^) as compared with nontreated control LNCaP and LNCaP-JIP-1 cells. When both cell types were cultured with the combination of DHT (10^−8^ mol l^−1^) and 10^−9^ mol l^−1^ of TPA, luciferase activity was reduced dramatically, compared with DHT containing cultures alone (Figure 7

). Thus, the DHT-stimulated upregulation and TPA-induced downregulation of ARE reporter activity in wild-type and LNCaP-JIP-1 cells were nearly identical.

## DISCUSSION

In this study, we showed that growth control of prostate cancer cells can be mediated through the JNK/c-Jun pathway, but androgen-responsiveness of these cells can be independent of this pathway. We established LNCaP cells stably transfected with JBD of JIP-1 (LNCaP-JIP-1 cells). When LNCaP-JIP-1 cells were cultured in the presence of TPA, the ability of JNK to phosphorylate c-Jun was effectively blocked. TPA-induced AP-1 transcriptional activity was abrogated, and TPA-induced growth arrest and apoptosis were attenuated. Further studies using the JNK inhibitor SP600125 confirmed the importance of the JNK/c-Jun/AP-1 signal pathway in TPA-induced growth arrest of LNCaP cells; SP600125 protected LNCaP cells from TPA-induced growth arrest. Recently, other investigators also reported the contribution of the JNK/c-Jun signaling to TPA-mediated apoptosis of LNCaP cells (Engedal *et al*, 2002); they transiently transfected JBD of JIP-1 in LNCaP cells to inhibit the JNK/c-Jun signal pathway. These transiently transfected cells were relatively resistant to TPA-induced apoptosis compared to untransfected control cells.

JIP-1 significantly attenuated the TPA-induced growth arrest and apoptosis in LNCaP cells; however, this effect was partial, indicating that other signal pathways must exist by which TPA causes decreased growth and increased apoptosis. One possible candidate is the Ras/Raf/ERK signal pathway. ERK is another member of the MAPK family which has an important role in regulating cellular proliferation and differentiation, induced by growth factors, cytokines, and TPA (He *et al*, 1999; Wang *et al*, 2000). TPA induced phosphorylation of ERK in LNCaP cells (Gschwend *et al*, 2000). In agreement with the previous study, we have found that TPA-exposure resulted in a slight phosphorylation of ERK in wild type LNCaP cells and a 3-fold phosphorylation of ERK in LNCaP-JIP-1 cells as measured by Western blot analysis (data not shown). ERK might function to compensate for the impaired JNK/c-Jun/AP-1 signal pathway in LNCaP-JIP-1 cells.

Previous studies showed that cross-talk existed between the nuclear hormone receptors and c-Jun/AP-1 (Jonat *et al*, 1990; Schule *et al*, 1990; Yang-Yen *et al*, 1990). c-Jun/AP-1 down-regulated the glucocorticoid receptor (GCR) activity by inhibiting the binding of GCR to the GCR response element. AR belongs to the nuclear hormone receptor family, and regulates target genes after activation of the receptor by binding of its ligand (Huang *et al*, 1999; Hisatake *et al*, 2000). TPA down-regulates AR transcriptional activity without decreasing levels of AR. Another study suggested that c-Jun played a role in this inhibition by binding to the DNA-binding domain of AR, resulting in the inhibition of binding of activated AR to its AREs (Sato *et al*, 1997). In our study, AR mediated transcriptional activity in LNCaP-JIP-1 cells was disrupted by TPA (10^−9^ M), even though phosphorylation and up-regulation of c-Jun levels and AP-1 reporter activity were effectively blocked in these cells, suggesting that other pathway(s) must play a role in the TPA-induced down-regulation of AR signal activity. As prostate cancer progresses, it often becomes independent of androgen control. Our study suggests that this may occur independent of the JNK/c-Jun pathway. The phosphoinositide 3-kinase (PI3K)/Akt pathway is constitutively active in LNCaP cells because of the loss of PTEN expression, and PI3K/AKT was shown to activate the AR signaling (Wu *et al*, 1998; Li *et al*, 2001). TPA might down-regulate PSA by inhibiting the PI3K/AKT signal pathway. The regulation of gene transcription by nuclear receptors requires the recruitment of a number of proteins characterized as coregulators, functioning either as co-activators or co-repressors (Glass and Rosenfeld, 2000; Rosenfeld and Glass, 2001). Further studies will explore if TPA could inhibit the recruitment of co-activators or promote the recruitment of co-repressors of AR.

**Figure 8:**
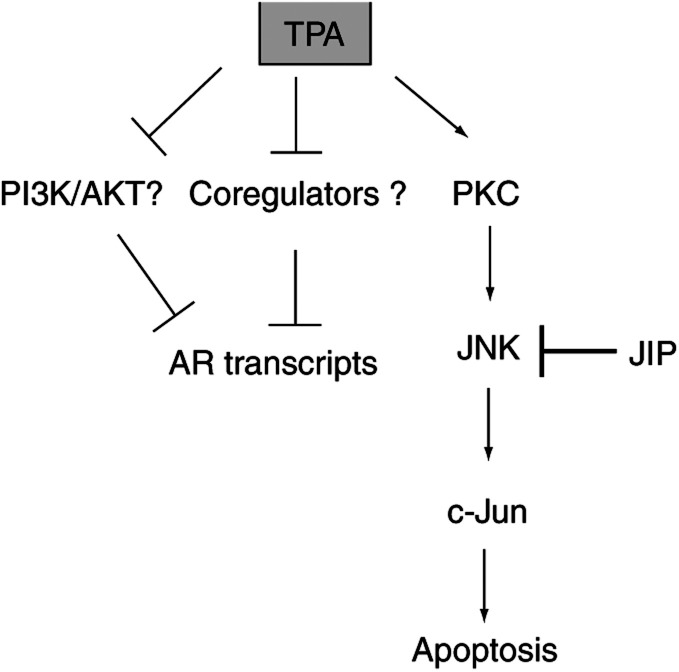
Mechanisms of action of TPA to induce apoptosis and inhibition of the AR signalling of LNCaP cells. 12-*0*-tetradecanoylphorbol-13-acetate activates the JNK/c-Jun signal pathway and induces apoptosis of LNCaP cells; however, inhibition of the AR signaling is independent of the JNK/c-Jun signal pathway. PKC, protein kinase C; JNK, c-Jun NH_2_-terminal kinase; JIP, JNK interacting protein; PI3K, phosphoinositide 3-kinase; AR, androgen receptor.

Taken together, these studies provide evidence that JNK helps to mediate the TPA-induced cell growth arrest and apoptosis in LNCaP human prostate cancer cells; and this effect might be mediated via phosphorylation of c-Jun. In contrast, we showed that the TPA-induced down-regulation of AR transcriptional activity was independent of the JNK/c-Jun/AP-1 signal pathway (Figure 8

). A recent phase I clinical study in individuals with relapsed/refractory hematological malignancies demonstrated the feasibility of TPA administration to humans resulting in therapeutic responses (Strair *et al*, 2002). Also, bryostatin 1, a related compound, has shown clinical activity when combined with high dose 1-beta-D-arabinofuranosylcytosine in individuals with relapsed/refractory acute leukemia (Cragg *et al*, 2002). Additional clinical studies in individuals with prostate cancer should be considered.
